# Pseudomonas aeruginosa persister cell formation upon antibiotic exposure in planktonic and biofilm state

**DOI:** 10.1038/s41598-022-20323-3

**Published:** 2022-09-27

**Authors:** Hiral Patel, Hasmatbanu Buchad, Devarshi Gajjar

**Affiliations:** Department of Microbiology and Biotechnology Centre, Faculty of Science, The Maharaja Sayajirao University of Baroda, Vadodara, Gujarat 390002 India

**Keywords:** Antimicrobial resistance, Pathogens

## Abstract

Persister cell (PC) is dormant, tolerant to antibiotics, and a transient reversible phenotype. These phenotypes are observed in *P. aeruginosa* and cause bacterial chronic infection as well as recurrence of biofilm-mediated infection. PC formation requires stringent response and toxin-antitoxin (TA) modules. This study shows the *P. aeruginosa* PC formation in planktonic and biofilm stages on ceftazidime, gentamicin, and ciprofloxacin treatments. The PC formation was studied using persister assay, flow cytometry using Redox Sensor Green, fluorescence as well as Confocal Laser Scanning Microscopy, and gene expression of stringent response and TA genes. In the planktonic stage, ceftazidime showed a high survival fraction, high redox activity, and elongation of cells was observed followed by ciprofloxacin and gentamicin treatment having redox activity and rod-shaped cells. The gene expression of stringent response and TA genes were upregulated on gentamicin followed by ceftazidime treatment and varied among the isolates. In the biofilm stage, gentamicin and ciprofloxacin showed the biphasic killing pattern, redox activity, gene expression level of stringent response and TA varied across the isolates. Ceftazidime treatment showed higher persister cells in planktonic growth while all three antibiotics were able to induce persister cell formation in the biofilm stage.

## Introduction

Persister cell (PC) is a non-growing and metabolically inactive cell, which lacks transcription, translation, and proton motive force. PC formation is reported to occur stochastically or under various conditions such as (i) upon antibiotic treatment (ii) nutrient deprivation and (iii) biofilms^1–3^. PC are responsible for chronic bacterial infections and relapse of biofilm infections^4–6^. Within the biofilm matrix, the PC escape from the attack of the host immune system and other harsh environmental conditions^4^. *Pseudomonas aeruginosa (P. aeruginosa)* is an opportunistic pathogen frequently causing chronic airway infections in patients with cystic fibrosis (CF), urinary tract infections (UTIs) and ventilator-associated pneumonia. It is also involved in persistent infections and biofilm-related infections^7,8^. The majority of in vitro data on PC has been focused on the exponential and stationary phase of *Escherichia coli* (*E. coli*).

In recent years studies have shown elements required for stringent response and persistence are ppGpp alarmone, SpoT, RelA, DksA, Lon, and Toxin-Antitoxin (TA) modules, and the regulatory role of these elements is well characterized in *P. aeruginosa* (PAO1)^9^. Antibiotics; ciprofloxacin, cefepime, colistin, and amikacin were shown to upregulate *relA* gene expression, apart from induction of *relA*, persister formation might also occur from different mechanisms with different antibiotics in the planktonic stage^10^. In *E. coli* the Lon protease degrades the antitoxin which leads to the accumulation of toxins leading to PC formation^11^. However, in *P. aeruginosa* the Lon protease activity is induced by aminoglycosides and necessary for virulence, motility, and biofilm formation^12^. In *P. aeruginosa,* biofilms increase the PC formation^13,14^, and the association of TA systems in persister formation is documented in type strain PAO1. Among which ParD/ParE^15^, HicA/HicB^16^, RelE/RelB^17^, and HigB/HigA^18^ TA systems are identified in *P. aeruginosa.* The well-studied type II TA system HigB/HigA influences swarming motility, biofilm formation, and virulence factors such as pyochelin and pyocyanin^18,19^. HigB reduces the biofilm formation through (i) reducing the intracellular c-di-GMP level through activation of c-di-GMP hydrolysis genes, which (ii) upregulates the expression of the type 3 secretion system (T3SS)^19^. Additionally, subsequent exposure to ciprofloxacin antibiotic results in PC formation via activation of RelA/SpoT and HigB/HigA TA system in biofilm^20^. Similarly, upon exposure to DNA gyrase inhibitor antibiotics the TA system ParD/ParE gets activated^15^.

The TA system and Lon protease have not been studied at supra-MIC concentration of antibiotics, and their significant role in biofilm tolerance as well as in the planktonic stage remains to be elucidated. In this study, we enumerate PC formation in the planktonic and biofilm stage with exposure to three different antibiotics (ceftazidime, gentamicin, and ciprofloxacin) in *P. aeruginosa*.

## Results

### Antimicrobial susceptibility of *P. aeruginosa* isolates

Supplementary Table S1 shows the MIC values of the *P. aeruginosa* clinical isolates: TP-10, ST-13, and PAO1 (as reference strain) (n = 3) for the respective antibiotics (ceftazidime, gentamicin, and ciprofloxacin). As per CLSI guidelines, PAO1 and ST-13 isolates were resistant to ciprofloxacin, while susceptible to ceftazidime and gentamicin antibiotics. The TP-10 isolate was susceptible to all antibiotics.

### Persister cell formation in the planktonic stage

**Figure 1:**
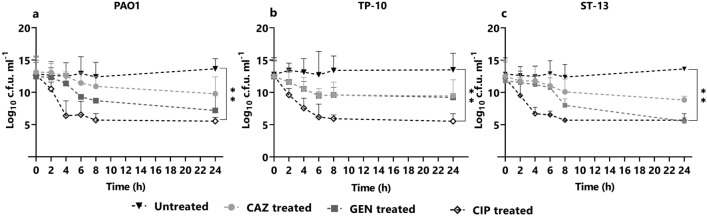
Planktonic time-kill curve assay. The planktonic stage of *P. aeruginosa:* (**a**) POA1, (**b**) TP-10, and (**c**) ST-13 were grown in LB and treated with CAZ, GEN, CIP at 5× MIC. At t = 2, 4, 6 8, and 24 h cells were plated for the viable count. Without treatment was used as a control. CAZ: ceftazidime; GEN: gentamicin, and CIP: ciprofloxacin. Statistical analysis was performed using one-way ANOVA, which was *P < 0.05, **P < 0.01.

*P. aeruginosa* isolates (PAO1, TP10, and ST13) were studied for PC formation using three bactericidal antibiotics: ceftazidime (Cephalosporin), gentamicin (aminoglycoside), and ciprofloxacin (fluoroquinolone) at 5X MIC. Figure 1 shows the time-kill curve assay for PAO1, TP10, and ST13 isolates using three antibiotics at 5X MIC concentration at indicated time points for 24 h. The biphasic killing pattern, which is a hallmark for PC, varied across the isolates. In the PAO1 strain, ceftazidime showed the least log reduction and formed a considerably higher cell survival fraction (9 log10 cfu/ml) (Fig. 1a). Gentamicin treatment formed 8 log10 cfu/ml, whereas ciprofloxacin treatment resulted in 6 log10 cfu/ml (Fig. 1a). Further in the TP-10 isolate, gentamicin and ceftazidime treatment formed a 9 log10 cfu/ml survival fraction (Fig. 1b) and ciprofloxacin treatment resulted in the cell survival fraction of 6 log10 cfu/ml. In the ST-13 isolate, on ceftazidime treatment the survival fraction was 9–10 log10 cfu/ml (Fig. 1c). On gentamicin and ciprofloxacin treatment the range of survival fraction was 5–6 log10 cfu/ml (Fig. 1c). The maximum log reduction was observed by ciprofloxacin treatment and least persister cells were formed compared to other antibiotic treatments.

### Cellular redox activity in the planktonic stage

**Figure 2:**
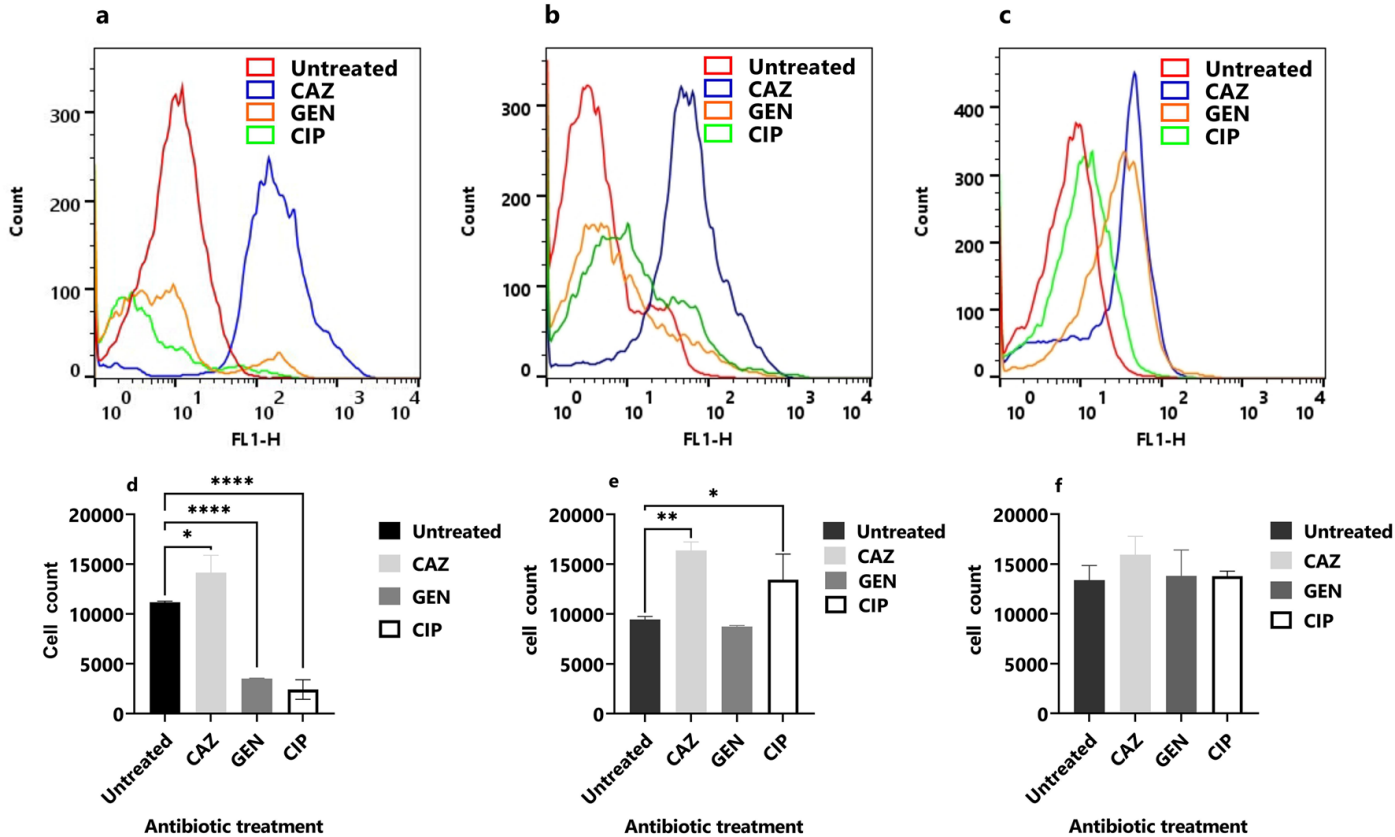
Cellular redox activity of planktonic cells. The flow cytometry of (**a**) PAO1, (**b**) TP-10 and (**c**) ST-13 isolate was done using RSG staining after 4 h of antibiotics treatment (CAZ, GEN, and CIP). Untreated was used as a control. (**d–f**) The quantitative data of RSG-positive cells (in PAO1, TP-10 and ST-13) using flow cytometry analysis after treatment with respective antibiotics were compared to the untreated control. CAZ: ceftazidime, GEN: gentamicin, and CIP: ciprofloxacin. The corresponding data represent the mean ± standard deviation. The experiment was performed in three biological triplicates for each strain. Statistical analysis was performed using a one-way ANOVA, which was *P < 0.05, and **P < 0.01, ***P < 0.001, ****P < 0.0001.

The cellular redox activity in the planktonic stage of isolates PAO1, TP-10, and ST-13 was done using RSG and PI staining. The RSG dye can easily penetrate bacteria and yield green fluorescence when reduced by bacterial reductases which can be correlated with cellular metabolic activities^21,22^. Figure 2a–c shows the flow cytometry of PAO1, TP-10, and ST-13 isolates with and without antibiotic treatments after 4 h. In PAO1 isolate, ceftazidime treatment leads to an increase in redox activity compared to untreated (Fig. 2a). Whereas, on gentamicin and ciprofloxacin treatment there was a decrease in redox activity (Fig. 2a). When compared to an untreated TP-10, ceftazidime treatment increased redox activity, followed by ciprofloxacin and gentamicin treatment (Fig. 2b). Ceftazidime treatment has increased redox activity in the ST-13 compared to untreated, followed by gentamicin and ciprofloxacin (Fig. 2c). Figure 2d–f shows the flow cytometry analysis of the number of RSG positive cells of antibiotic-treated and untreated. In PAO1 there is a significant increase in redox activity on ceftazidime treated compared to untreated. While redox activity was decreased on gentamicin and ciprofloxacin (Fig. 2d). In the TP-10, there is a significant difference in RSG fluorescence after ceftazidime and ciprofloxacin treatment compared to untreated cells (Fig. 2e). While there was no significant difference observed in the ST-13 isolate (Fig. 2f).

### Fluorescent microscopy of the planktonic stage

**Figure 3:**
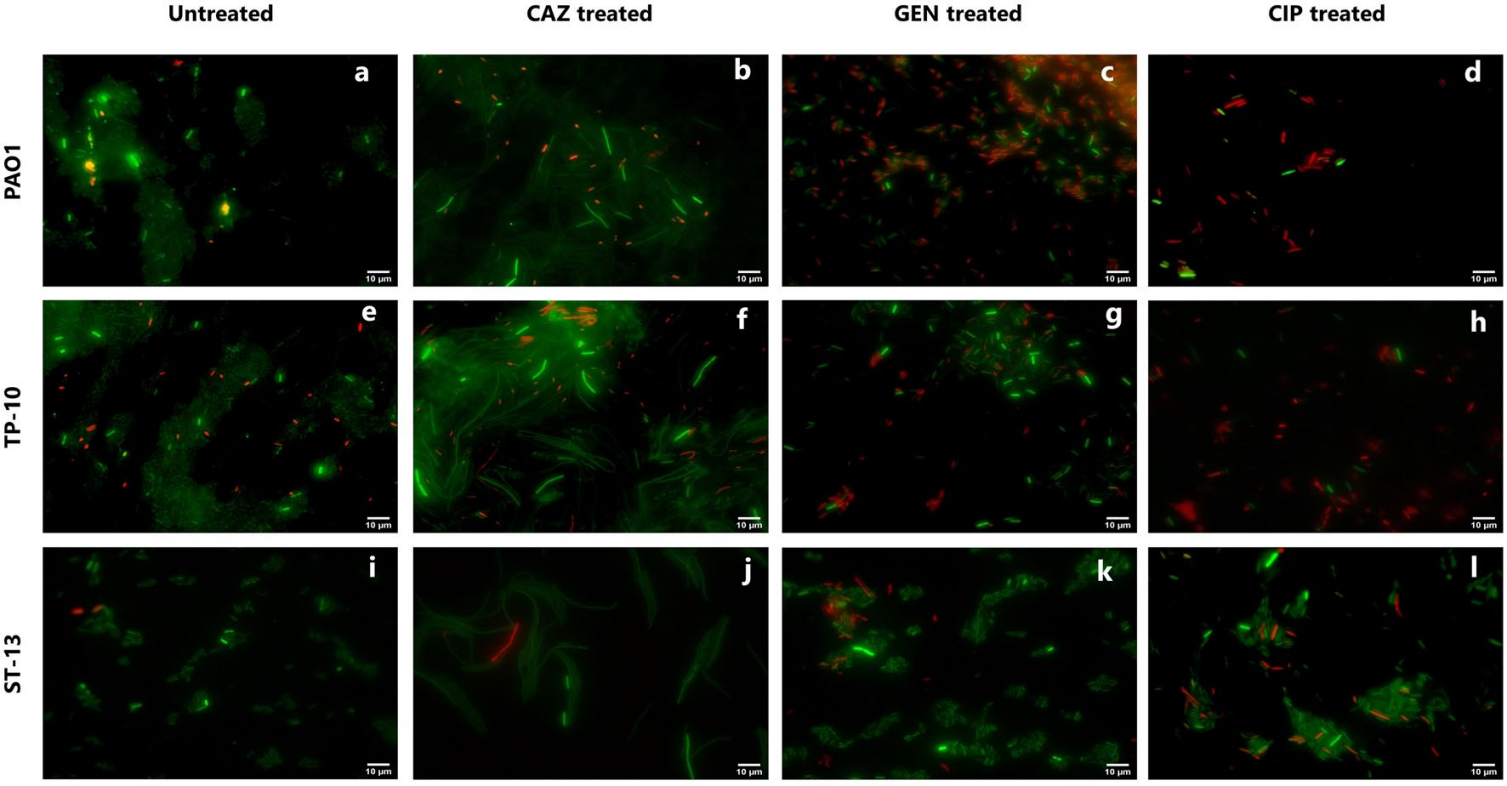
Fluorescence Microscopy of PCs in the planktonic stage. The representative images of PAO1, TP-10, and ST-13 isolates, after 4 h of (**a,e,i**) without antibiotic treatment and with (**b,f,j**) CAZ (**c,g,k**) GEN (**d,h,l**) CIP antibiotic treatments, which were grown in LB. Before staining the cells were washed to remove antibiotics and stained with RSG and PI dyes. (**a,e,i**) Untreated cells were used as control. Cellular redox activity was measured by RSG staining. CAZ: ceftazidime, GEN: gentamicin, and CIP: ciprofloxacin. Images were analysed using Fiji software.

Figure 3 shows the fluorescence microscopy images of PC formation after 4 h of antibiotic treatment at 5X MIC. In PAO1 isolate, ceftazidime treatments lead to the filamentous form of cells, while gentamicin and ciprofloxacin had rod-shaped cells (Fig. 3b–d). In TP-10 isolate, on ceftazidime treatment, elongated cells were observed as PAO1, while on gentamicin and ciprofloxacin treatment rod-shaped cells were observed (Fig. 3f–h). Apart from this, the high redox-active cells were present on ceftazidime and gentamicin treatments in the PAO1 (Fig. 3b,c), and TP-10 (Fig. 3f,g). On the ciprofloxacin treatment, there were fewer high redox-active cells and more number of cells were killed (Fig. 3d,h). While in ST-13, the ceftazidime antibiotic leads to elongation of cells with high redox activity (Fig. 3j). The gentamicin and ciprofloxacin treatment had rod shape cells, where the number of high redox-active cells was less in number (Fig. 3k,l).

### Gene expression in the planktonic stage

To gain a better understanding of the mechanism behind the *P. aeruginosa* (PAO1, TP-10, and ST-13) persistence in the planktonic stage upon ceftazidime, gentamicin, and ciprofloxacin treatment, the gene expression was studied for antibiotic tolerance: stringent response genes (*relA, spoT,* and *lon)* and the toxin-antitoxin genes (*higA* and *higB*) involved in PC formation.

**Figure 4:**
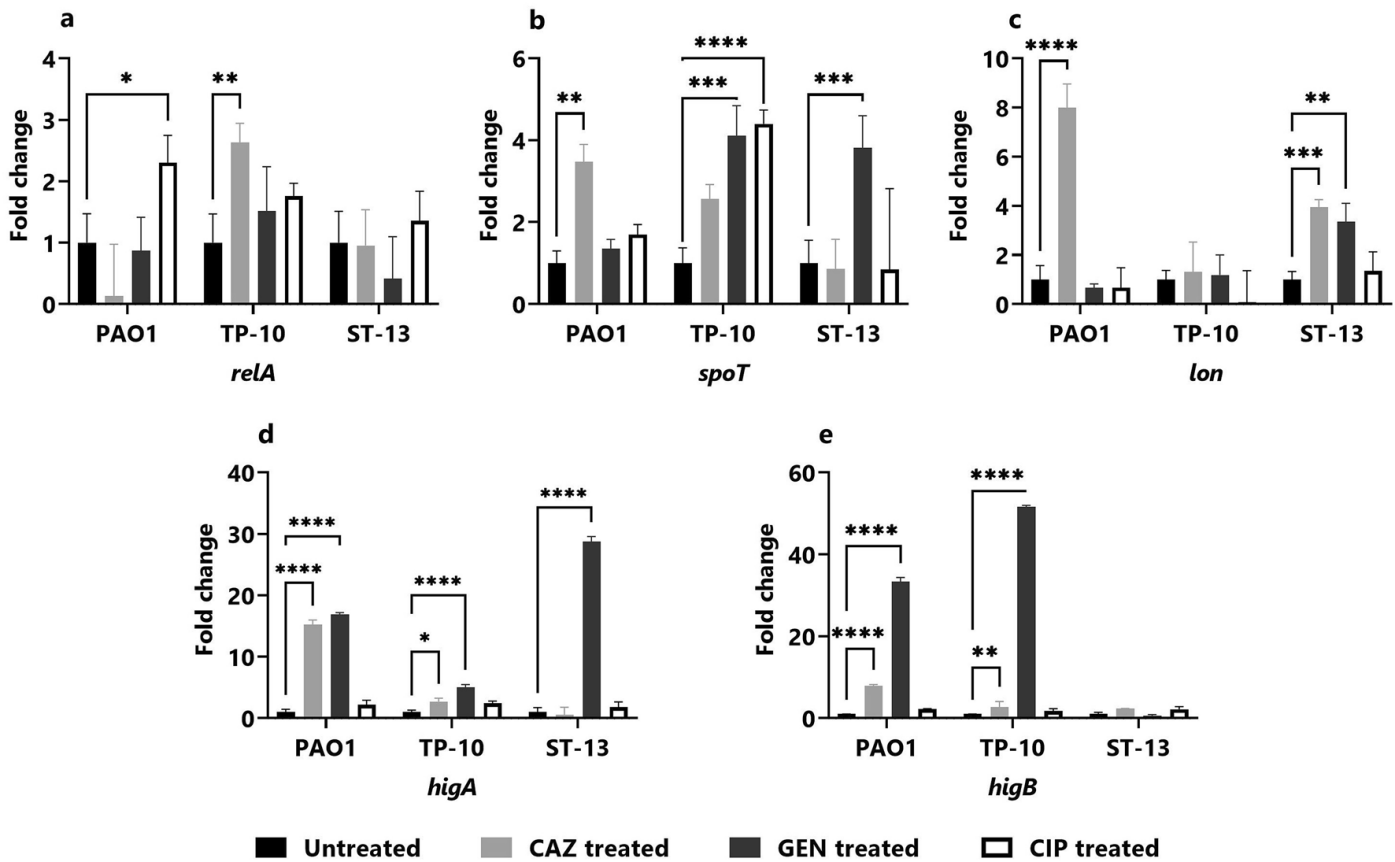
Gene expression studies in the planktonic stage. The gene expression of stringent response genes: (**a**) *relA*, (**b**) *spoT*, and (**c**) *lon* as well as toxin–antitoxin genes; (**d**) *higA* and (**e**) *higB* was studied in PAO1, TP-10 and ST-13 isolate after 4 h of antibiotic (CAZ, GEN, and CIP) treatment. The gene expression was normalized with untreated control. CAZ: ceftazidime, GEN: gentamicin, and CIP: ciprofloxacin. The experiment was performed in three biological experiments for each strain. Data represent the mean ± standard deviation of fold change. The Statistical analysis was performed using the two-way ANOVA, which was *P < 0.05, **P < 0.01, ***P < 0.001, and ****P < 0.0001.

Figure 4 depicts the variation of the gene expression across isolates (PAO1, TP-10, and ST-13) in the planktonic stage, with antibiotic treatments showing upregulation of genes as compared to untreated. The *relA* gene expression was 2.30 ± 0.44-fold higher on ciprofloxacin treatment in PAO1, and 2.63 ± 0.32-fold higher on ceftazidime treatment in TP-10 (Fig. 4a). Further, *spoT* gene expression was 3.44 ± 0.43-fold upregulated on ceftazidime treatment in PAO1 and 4.11 ± 0.72 and 4.40 ± 0.34-fold higher on gentamicin and ciprofloxacin treatment, respectively in TP-10 (Fig. 4b). While in ST-13 upregulation of *spoT* gene was 3.81 ± 0.78-fold higher on gentamicin treatment (Fig. 4b). The *lon* gene expression was increased by 7.99 ± 0.96-fold on ceftazidime treatment in PAO1, and 3.94 ± 0.30 and 3.36 ± 0.074-fold increase in response to ceftazidime and gentamicin treatment, in ST-13 (Fig. 4c). Additionally, the expression of *higA* gene was significantly upregulated by 15.26 ± 0.70 and 16.88 ± 0.30-fold on ceftazidime and gentamicin treatment, respectively, in PAO1 (Fig. 4d). Also, the *higA* gene was a 2.72 ± 0.52 and 5.05 ± 0.42-fold increase on ceftazidime and gentamicin treatment in TP-10 and 28.77 ± 0.80-fold high on gentamicin treatment in ST-13 (Fig. 4d). The expression of *higB* gene was 7.90 ± 0.33 and 33.33 ± 1.02-fold upregulated on ceftazidime and gentamicin treatment in PAO1, while 2.74 ± 1.3 and 51.63 ± 0.35 upregulated on ceftazidime and gentamicin in TP-10 (Fig. 4e).

### Persister cell formation in the biofilm stage

**Figure 5:**
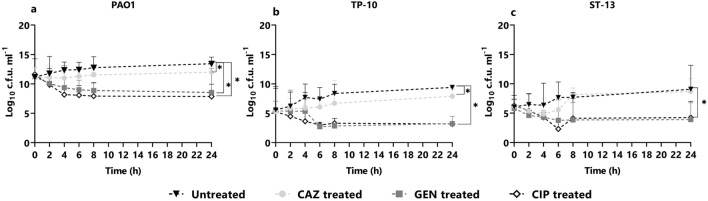
Biofilm time-kill curve assay. The biofilm formed for 24 h of (**a**) POA1, (**b**) TP-10, and (**c**) ST-13, grown in MHB, were treated with CAZ, GEN, CIP at 5× MIC concentration. At indicated time points of t = 2, 4, 6 8, and 24 h cells were plated for the viable count. Without treatment was used as a control. CAZ: ceftazidime, GEN: gentamicin, and CIP: ciprofloxacin. Statistical analysis was performed using one-way ANOVA, which was *P < 0.05, **P < 0.01.

Biofilm formation was analyzed for all three isolates using crystal violet assay, in which PAO1 and ST-13 were found to be strong biofilm producers and TP-10 was a weak biofilm producer (Supplementary Fig. S1). To analyze the PC formation in biofilm conditions, a biofilm time-kill curve experiment was performed for PAO1, TP-10, and ST-13 isolates against three antibiotics as shown in Fig. 5a. The mean inoculum varied between isolates, ranging from 5–6 log10 cfu/ml for TP-10 and ST-13 to 11–12 log10 cfu/ml for PAO1. After 24 h of ceftazidime treatment, all isolates were regrown to the same cfu/ml as the growth control (Fig. 5a–c). In all three isolates, the biphasic kill curve was observed for gentamicin and ciprofloxacin antibiotic treatment (Fig. 5a–c). The biphasic curve showed an initial log decrease in PAO1 biofilm after treatment with gentamicin and ciprofloxacin, followed by 8–9 log10 cfu/ml and 8 log10 cfu/ml cell surviving fractions. In TP-10 biofilm, approximately 2–3 log10 cfu/ml cell survival fractions were formed in biphasic kill curves on gentamicin and ciprofloxacin treatment. In addition to gentamicin and ciprofloxacin treatment, 3–4 log10 cfu/ml cell survival fractions were observed in ST-13 biofilm.

### Cellular redox activity in the biofilm stage

**Figure 6:**
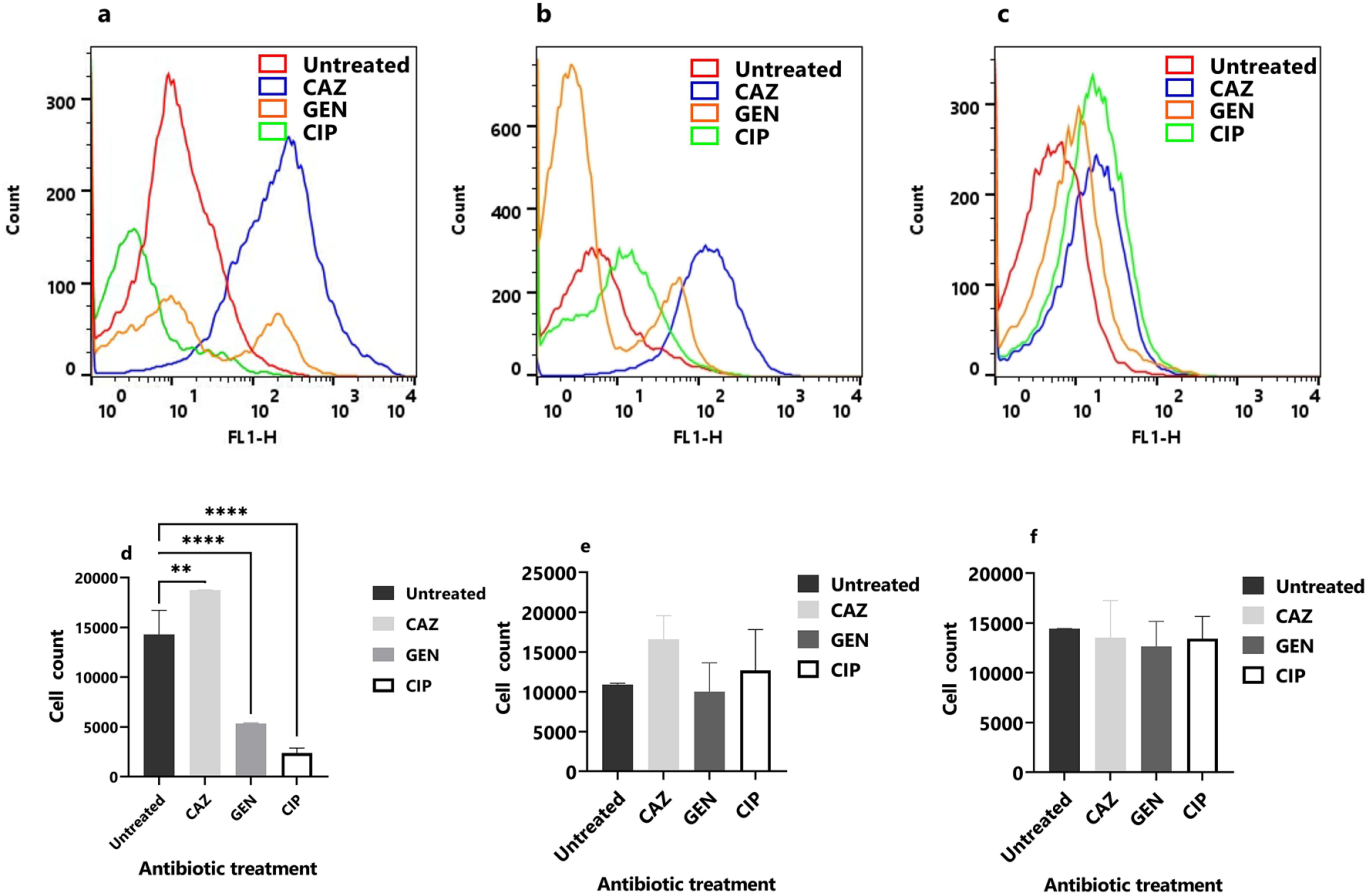
Cellular redox activity in the biofilm stage. The flow cytometry of (**a**) PAO1 (**b**) TP-10 and (**c**) ST-13 biofilms was done using RSG and PI staining after 4 h of antibiotics treatment (CAZ, GEN, and CIP). Untreated was used as a control. (**d–f**) The quantitative data of RSG-positive cells (in PAO1, TP-10 and ST-13) using flow cytometry analysis after treatment with respective antibiotics were compared to the untreated control. CAZ: ceftazidime, GEN: gentamicin, and CIP: ciprofloxacin. The corresponding data represent the mean ± standard deviation. The experiment was performed in three biological triplicates for each strain. Statistical analysis was performed using a one-way ANOVA, which were *P < 0.05, and **P < 0.01, ***P < 0.001, and ****P < 0.0001.

The cellular redox activity in the biofilm stage after 4 h of antibiotic treatments (Ceftazidime, gentamicin, and ciprofloxacin) was studied using flow cytometry (Fig. 6). In PAO1, ceftazidime treatment leads to an increase in redox activity compared to control (Fig. 6a). Whereas, there was no increase in redox-active cells on gentamicin and ciprofloxacin treatments (Fig. 6a). In the TP-10 biofilm, the ceftazidime treatment showed high redox-active cells followed by gentamicin and ciprofloxacin compared to untreated (Fig. 6b). While in ST-13 biofilm, high redox-active cells were observed on ceftazidime treatment followed by ciprofloxacin and gentamicin (Fig. 6c). Figure 6d–f depicts flow cytometry analysis of RSG positive cells after antibiotic treatments. In PAO1 biofilm, ceftazidime treatment resulted in a high number of redox-active cells than untreated control (Fig. 6d). While a decrease in redox-active cells was observed on gentamicin and ciprofloxacin treatments (Fig. 6d). In TP-10 and ST-13 biofilm, no significant difference in redox activity was observed compared to untreated control (Fig. 6e,f).

### CLSM microscopy of the biofilm stage

**Figure 7:**
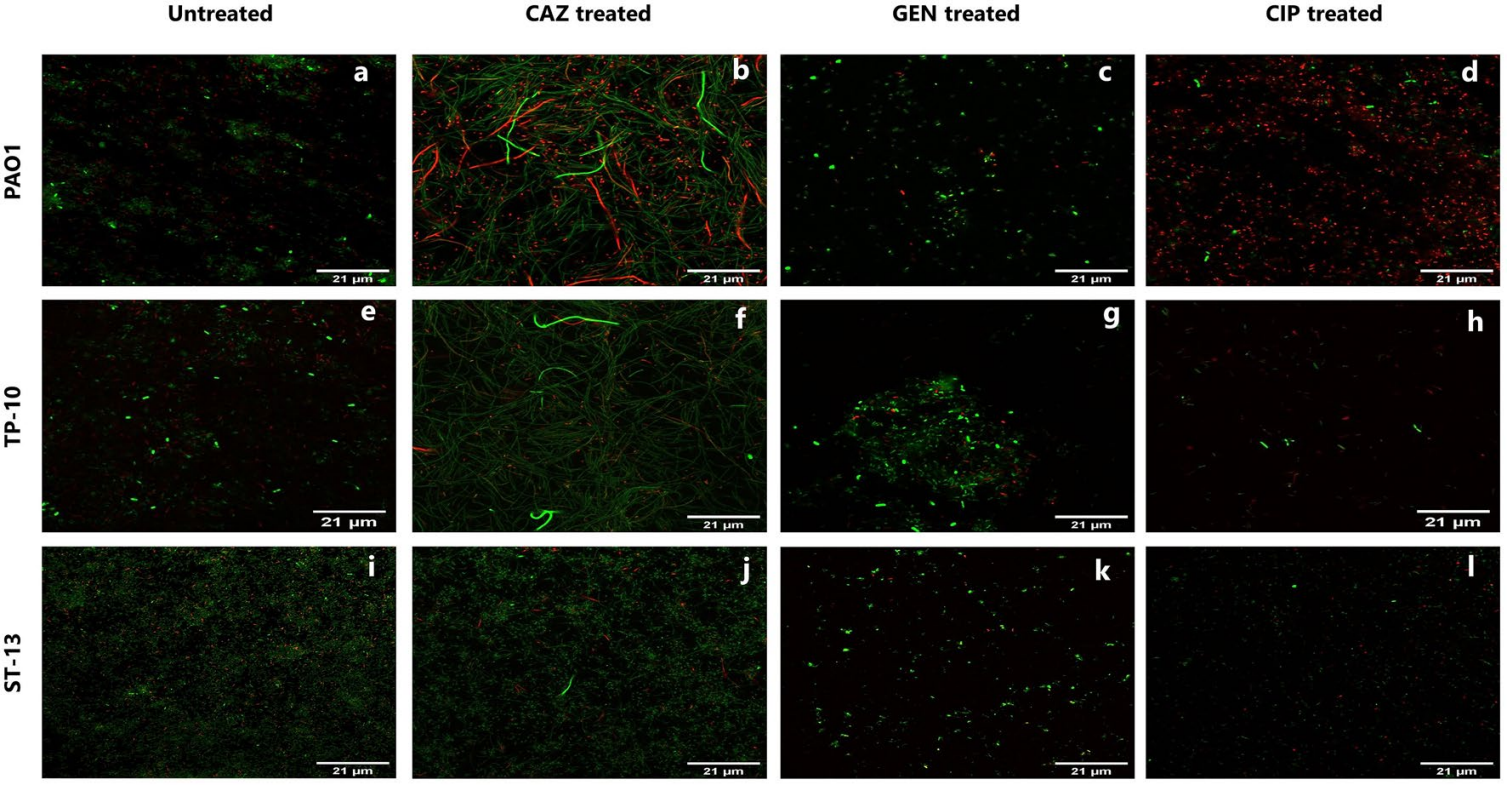
CLSM of PC in the biofilm stage. The representative images of PAO1, TP-10, and ST-13 biofilm, grown in MHB, after 4 h of (**a,e,i**) without antibiotic treated and antibiotic treatment with (**b,f,j**) CAZ, (**c,g,k**) GEN, and (**d,h,l**) CIP. Biofilm was rinsed with 0.85% NaCl to remove any residue of antibiotics and staining was done using RSG and PI dyes. CAZ: ceftazidime, GEN: gentamicin, and CIP: ciprofloxacin. Images were analysed using Fiji software.

The cellular redox activity within the biofilm formed by PAO1, TP-10, and ST-13 after 4 h of treatment with antibiotics were visualized by CLSM using RSG and PI staining (Fig. 7). Biofilms formed by three isolates showed varying thickness. In PAO1 biofilm, treated had high redox-active cells compared to untreated one (Fig. 7a-d). Ceftazidime treatment had elongated cells; however, the high redox-active cells were observed less (Fig. 7b). On gentamicin and ciprofloxacin treatment rod-shaped high redox-active cells were observed as well as disrupted biofilm and dead cells were high in number in ciprofloxacin treated PAO1 biofilm (Fig. 7c,d). On ceftazidime treatment, elongation of cells observed with few redox-active cells was observed in TP-10 biofilm compared to the untreated (Fig. 7e,f). On gentamicin treatment, rod-shaped high redox-active cells were observed in TP-10 biofilm (Fig. 7g). Whereas on ciprofloxacin treatment TP-10 biofilm was disrupted and fewer higher redox active cells were observed (Fig. 7h). ST-13 isolate had thick biofilm in untreated, ceftazidime treatment had very less elongation of cells with a smaller number of redox-active cells (Fig. 7i,j). On gentamicin treatment had rod-shaped high redox-active cells and biofilm was disrupted in ST-13 biofilm (Fig. 7k). While ciprofloxacin treatment had less number of cells compared to untreated and biofilm was disrupted having fewer redox-active cells (Fig. 7l).

### Gene expression in biofilm stage

**Figure 8:**
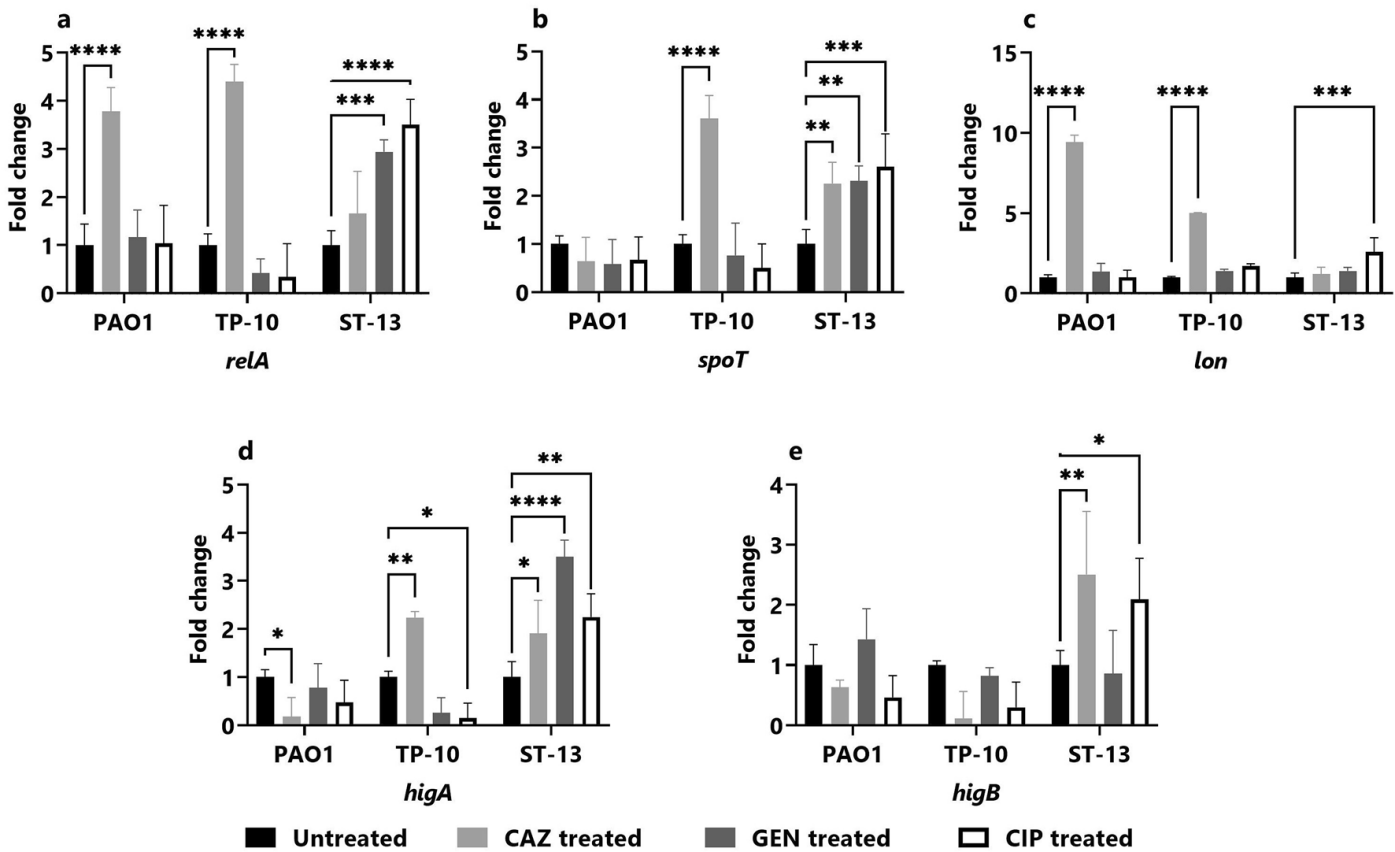
Gene expression studies in the biofilm stage. The gene expression of stringent response genes: (**a**) *relA*, (**b**) *spoT*, and (**c**) *lon* as well as toxin–antitoxin genes; (**d**) *higA* and (**e**) *higB* was studied in PA01, TP-10, and ST-13 biofilms after 4 h of antibiotic (CAZ, GEN, and CIP) treatment. Prior to it, the biofilm was formed for 24 h in six well plates. The gene expression was normalized to the untreated control. CAZ: ceftazidime, GEN: gentamicin, CIP: ciprofloxacin. The three biological experiments were performed for each strain. The corresponding data represents the mean ± standard deviation of fold change. The statistical analysis was performed using the two-way ANOVA, which was *P < 0.05, **P < 0.01, ***P < 0.001, and ****P < 0.0001.

After 4 h of antibiotic treatment, the gene expression of stringent response genes (*relA, spoT,* and *lon*), and the toxin-antitoxin genes (*higA* and *higB*) involved in PCs formation was examined, and compared with untreated (Fig. 8). The expression of *relA* gene was elevated by 3.78 ± 0.50-fold and 4.40 ± 3.50-fold on ceftazidime treatment, in PAO1 and TP-10 respectively (Fig. 8a). The expression of *relA* gene was upregulated by 2.94 ± 0.25 and 3.50 ± 0.52-fold on gentamicin and ciprofloxacin treatment in ST-13 (Fig. 8a). The *spoT* gene was increased by 3.61, ± 0.47-fold on ceftazidime treatment in TP-10, and 2.31 ± 0.311 and 2.60 ± 0.68-fold increased on gentamicin and ceftazidime treatment in ST-13 (Fig. 8b). The *lon* gene was 9.45 ± 0.42 and 5.01 ± 0.02-fold higher on ceftazidime treatment in PAO1 and TP-10, respectively (Fig. 8c). Further, *higA* gene was upregulated by 2.24 ± 0.12-fold on ceftazidime treatment in TP-10 and 1.91 ± 0.68, 3.50 ± 0.34, and 2.24 ± 0.48-fold in all three-antibiotic treatment in ST-13 (Fig. 8d). Additionally, the expression of *higB* gene was 2.50 ± 1.05, and 2.09 ± 0.68-fold high on ceftazidime and ciprofloxacin treatment in ST-13 (Fig. 8e).

## Discussion

The persistence phenomenon is a type of phenotypic switch that allows the subpopulation of bacteria to survive under antibiotic exposure and is associated with recurrent infections and antibiotic treatment failure. PC formation is a complex survival strategy and depends upon various factors that include growth phase, expression of stringent response genes, toxin-antitoxin systems, quorum sensing, and SOS response^13^. Many studies have documented PC formation upon antibiotic exposure with respect to the type strains of *P. aeruginosa;* PAO1 and PA14^14,17,20^. The present study highlights the PC formation in response to exposure of three antibiotics belonging to different classes: ceftazidime, gentamicin, and ciprofloxacin in PAO1, TP-10, and ST-13 strains under two physiological conditions (planktonic and biofilm stage). The effect of three antibiotics that lead to PC formation was analyzed by measuring the redox activity and expression of stringent response as well as toxin-antitoxin genes in planktonic and biofilm conditions.

In the planktonic stage, ceftazidime showed a biphasic kill-curve in all isolates; showing the presence of PCs. However, the effect of gentamicin and ciprofloxacin was diverse across the isolates (Fig. 1). Further, the presence of PCs was confirmed using RSG staining where a difference in cellular redox activity was observed between the strains, and high redox activity was seen under ceftazidime treatment followed by ciprofloxacin and gentamicin. The difference in redox activity observed among isolates is due to the difference in survival fractions observed in cfu/ml data. A similar study showed more survival fraction in β-lactam (carbenicillin) treatment compared to fluoroquinolone (ofloxacin) and an aminoglycoside (tobramycin)^23^. Reduced survival rate upon ciprofloxacin treatment that kills non-growing cells also showed variability across the clinical isolates of *P. aeruginosa*^10,24^. In this study, microscopy of antibiotic treated planktonic cells showed filamentous PCs with high redox activity upon ceftazidime treatment (Fig. 3). The ceftazidime treatment causes filamentous formation by directly binding to Penicillin Binding Protein 3 (PBP3) and inhibiting septal division after doubling of cells^25^. Recent studies on *E. coli* showed that ampicillin treatment leads to filamentous PC formation with high reactive oxygen species (ROS)^26^, while rifampicin treatment leads to rod-shaped and metabolically inactive^27^ and ofloxacin treated persister were also elongated^28^.

Stringent response (*relA* and *spoT*) is induced upon antibiotic exposure leading to increase in alaromne of (p)ppGpp which arrests the cell growth (by directly interacting with RNA polymerase and inhibiting the transcription) and provides fitness advantage^9,29^. Together with stringent response, the TA system activation also plays an important role in PC formation by reducing the cellular metabolism. In this study, enhanced expression of *relA*, *spoT* as well as *higA* and *higB* was observed, but the expression varied across the isolates (PAO1, TP-10, and ST-13) and changed with respect to antibiotic exposure (Fig. 4). A previous study has reported that *relA* gene was upregulated on treatment with colistin, amikacin, ciprofloxacin, and cefepime in the mid-exponential phase of *P. aeruginosa* clinical isolates^10^. Expression of *higA* and *higB* was also seen to be differentially responding upon exposure to different antibiotics amongst the isolates (Fig. 4). The elevated level of *higB* expression and its role in increasing the fraction of PCs was reported for ciprofloxacin^30^. TA affects the DNA gyrase activity, modulates the ribosome maturation, reduces the metabolism and growth to support the PC formation^31^. Upon gentamicin treatment, the HigA antitoxin is degraded by Lon protease which enhances the expression of *higB* and *mvfR,* and thus increases virulence gene expression^12,30,32^. Our results show high expression of *relA*, *spoT* along with the *higA* and *higB* in the susceptible isolate (TP10) while *spoT* and *lon* in the resistant isolate (ST13). We hypothesize, PC formation occurred through activation of stringent response and TA system in susceptible isolate. Whereas in resistant isolate the PC formation is regulated by *spoT* and *lon.*

Another aspect of this study is to analyse the PC formation in biofilm with regard to antibiotic treatments. Biofilm is difficult to eradicate owing to its high tolerance towards antibiotics^7,33^, and its transition towards persister phenotype in *P. aeruginosa* is not fully elucidated. These antibiotic-induced dormant cells are one of the reasons for biofilm tolerance^34^ and the cause of recurrent biofilm-mediated infection. Studies on *P. aeruginosa* biofilm have been reported by using CLSM with Syto 9 (stains live cells) and PI (stains dead cells) dye where β-lactam treatment showed the filamentous formation of cells, disruption of biomass, and regrowth of biofilm^35,36^. Further, aminoglycosides resulted in the decline of biofilm biomass, and the live cells were observed in the inner layer of biofilm^35^. Tolerance towards aminoglycosides was reported due to oxygen limitation, low metabolic activity within the inner layers of biofilm, and slow diffusion of antibiotics through biomass^37–39^. While fluoroquinolone treatment, particularly ciprofloxacin resulted in a deep decline in biofilm structure, dead cells, and disruption of biofilm biomass^20^. Ciprofloxacin is associated with the formation of vacuoles, and cell lysis, which results in the extrusion of intracellular components^40^. In the present study, firstly the thickness of biofilms formed by the three isolates varied and was different even between the strong biofilm forming isolates (PAO1and ST13) (Fig. S1). The reason for this is due to the varying adhesion and twitching motility behaviour^41^. Also, when antibiotics with a different mode of action were used it showed unpredictability in the survival amongst the isolates. This is attributed to the varying degree of penetration of antibiotics due to the different biofilm thickness. Wide variation in PCs formation amongst isolates in response to different antibiotics was previously reported for other bacteria as well^34,42,43^. This indicates that persistence not only depends upon the class of antibiotics but it also responds differently because of heterogeneity amongst the isolates and growth phase.

In the biofilm stage the expression of the stringent response, as well as toxin-antitoxin genes, was different across isolates. Ceftazidime treatment showed significant upregulation in stringent response as well as toxin-antitoxin genes followed by gentamycin (Fig. 8). Biofilms generated by a *P. aeruginosa* ∆*relAspoT* double knockout mutant were found to be more susceptible to fluoroquinolones, meropenem, colistin, and gentamicin^29^. In the present data, inspite of increased *lon* expression the expression of *higA* was reduced and there was no change in expression of *higB* in PAO1 biofilm upon ceftazidime treatment (Fig. 8c–e). This indicate that transcriptional level of *higA* and *higB* are additionally regulated by unknown transcriptional regulator in biofilm stage and similar observation was reported by Song et al. 2020^44^. Additionally, beta-lactam (ceftazidime) did not affect the persister level via Lon protease activation^45^. Further, when *P. aeruginosa* biofilm is exposed to gentamicin, Lon protease is activated, which degrades the antitoxin HigA, and transcription of toxin *higB* was reported to increase^12,30^. Guo et al. 2019 showed upregulation of *higB* gene after 30 min of exposure to gentamicin, due to degradation of HigA protein by Lon protease in late stationary phase cells^32^. Stringent response genes *relA*, *spoT* and *lon* as well as toxin-antitoxin gene *higBA* were elevated upon ciprofloxacin treatment when analysed after 1 h of exposure^20^. Same study also reported stable gene expression in of stringent response after 4 h and no upregulation of *higB* gene. This significant difference in gene expression amongst the isolates suggests that biofilm persistence is highly strain-specific, depending upon the degree of biofilm formation, as well as the level of antibiotic exposure. This study provides the preliminary evidence on multiple dynamics of PC formation in clinical isolates. However, further complete transcriptomics studies will give better understanding of key molecular mechanism of PC formation.

This study shows that in the planktonic stage ceftazidime treatment gave rise to PCs to a greater extent than the other tested antibiotics, while on the other hand, in the biofilm stage gentamicin and ciprofloxacin gave rise to PCs. Taken together this study suggest that activation of stringent response and type II TA system differently contributed to the PC formation in response to different antibiotics.

## Material and method

### Bacterial strains, growth conditions, and antibiotics used for the study

In this study, UTIs causing *P. aeruginosa* isolates (TP-10 and ST-13) and PAO1 strain were used. Bacterial isolates were routinely sub-cultured on Pseudomonas Isolation Agar (PIA). Minimum Inhibitory Concentration (MIC) was determined in Muller Hinton Broth (MHB). Three antibiotics: ceftazidime (cell wall inhibitor), Gentamicin (protein inhibitor), and Ciprofloxacin (DNA gyrase inhibitor) were used for this study.

### Minimum inhibitory concentration (MIC) determination

In the 96-well microtiter plate, the MIC was determined according to the CLSI guideline^46^. Briefly, 100 µl of Mueller Hinton Broth (MHB) was dispensed into the microtiter plate, followed by 50 µl of 4× desired antibiotic (dissolved in MHB) concentration was added to each well. Thereafter, 50 µl of 1:100 diluted culture (0.08 OD at 600 nm) was added to each well and incubated at 37 °C for 24 h under static conditions. Additionally, a growth control was grown without antibiotics. The MIC was concluded at the lowest concentration of three antibiotics that inhibited visible growth. The experiment was performed in biological triplicate for each strain.

### Planktonic persister assay

Cell viability count was measured after antibiotic treatment to determine the presence of PC formation. The PCs formation was determined for PAO1, TP-10, and ST-13 as reported previously^47^. To determine PCs formation in the planktonic stage, 3 ml of 0.1 OD at 600 nm culture was treated with 5× MIC concentration of antibiotics (ceftazidime, gentamicin, and ciprofloxacin) and kept on agitation at 250 rpm for 24 h in Luria Bertini (LB) medium. Colony-forming units (c.f.u.) were determined at time points of t = 0 h, 2 h, 4 h, 6 h, 8 h, and 24 h by sampling one strain per tube. Briefly, 100 µl of culture was sampled; washed with Phosphate Buffer Saline (PBS); serially diluted followed by spreading on Luria Agar (LA) plate, and incubated at 37 °C for 24 h for c.f.u. determination. The experiment was performed in biological triplicates.

### Cellular redox activity in the planktonic stage

The cellular redox activity was studied after 4 h of antibiotic treatment at 5× MIC using RSG (Redox Sensor Green) and PI (Propidium iodide) staining. Briefly, the overnight grown culture was diluted to 0.1 OD at 600 nm in 3 ml LB and treated with antibiotic treatments as per the above-mentioned protocol. After 4 h of antibiotic treatment, 3 ml of sample was aliquoted, washed with PBS twice, and resuspended in 300 µl of PBS. For flow cytometry, the cells were stained using RSG (1 µl) and PI (1 µl of 1:100 diluted) dyes (BacLightTM RedoxSensorTM Green Vitality Kit Thermo Fisher Scientific Inc., Waltham, MA, USA) for 10 min in dark condition. No antibiotic treatment was used as a control. The flow cytometry (Becton Dickinson FACS Calibur, New Jersey, United States) was performed for the detection of fluorescence signals using FLI and FL3 channels. The RSG-positive cells were plotted in a histogram against the FL1 channel. The data were analysed using three independent biological triplicates^27^.

### Fluorescence microscopy

PCs formation was observed after 4 h of antibiotic treatment according to the procedure outlined above. After 4 h of incubation, 1 ml of sample was aliquoted; PBS wash was given to remove any residual antibiotic. The sample was resuspended in 100 µl of PBS and stained with 1 µl RSG and 1 µl of 1:100 diluted PI concentration. Thereafter, 10 µl of the sample was spotted on a 1% agarose pad and covered with a 22 mm coverslip. The cells were viewed under a 100X oil immersion fluorescence (BX 51 Olympus microscope, Japan) microscope. For dead cells, cells were treated with ethanol and as a control, no antibiotic treatment was used^27^. The experiment was performed in triplicates.

### Gene expression in the planktonic stage

After 4 h of antibiotic treatment with 5X MIC, 3 ml of cells were centrifuged for 5 min at 8000 rpm and cells were washed in PBS to remove antibiotics and resuspended in 300 µl PBS. Total RNA was extracted using the Nucleospin RNA kit (Macherey Nagel, Hoerdt, France) and DNase treatment was performed as per the manufacturer’s instruction. On a 2% gel, RNA integrity was verified and quantified using nanodrop (Thermo Fisher Scientific, Waltham, MA, USA). The First-strand cDNA synthesis was performed with a prime 1st Strand cDNA synthesis kit (Takara, Bio in, Japan) per protocol. The stringent response genes (*relA, spoT,* and *lon*), as well as toxin-antitoxin genes (*higA* and *higB*), were studied using the primer listed in Supplementary Table S2. The PCR cycling conditions were as follows: 95 °C for 5 min and 40 cycles of 95 °C for 30 s, 58 °C for 30 s, 72 °C for 30 s. Relative quantification was carried out from three independent biological replicates. Data were normalized to untreated and fold changes were calculated according to the 2^–ΔΔCt^ method^48^.

### Biofilm model

In the six well polystyrene plates, 3 ml of bacterial culture (0.01 OD at 600 nm) was added and incubated at 37 °C for 24 h in static conditions. The next day, planktonic suspensions were removed and biofilms were gently rinsed with 1 ml of 0.85% NaCl to remove planktonic cells. Thereafter, 3 ml of fresh MHB was added and challenged with a 5× MIC concentration of antibiotics for 24 h^20^.

### Biofilm quantification

According to Stepanovic et al. 2004, the biofilm was grown in the 96-well plates. Briefly, 20 µl of overnight culture (0.2 OD at 600 nm) and 230 µl of LB were kept at 37 °C for 24 h of incubation. Only LB was used as blank control. The next day, biofilm was rinsed with 0.85% saline; fixed with methanol for 15 min; stained with 0.1% crystal violet (CV) for 15 min; CV was removed and rinsed with distilled water (DW) twice; air-dried and a further 33% glacial acetic was used to dissolve the attached CV. According to Stepanović et al. 2004, biofilm formation was quantified at 570 nm and categorized into strong, moderate, and weak based on cut-off OD (ODc) value (three standard deviations above the mean OD of the negative control)^49^. Isolates were categorized as: no biofilm producer (OD ≤ ODc), weak biofilm producers (ODc < OD < 2 × ODc), moderate biofilm producers (2 × ODc < OD < 4 × ODc), and strong biofilm producers (4 × ODc < OD). The experiment was performed in three biological replicates.

### Biofilm persister assay

In this study, the biofilm was formed as mentioned above for 24 h and treated with different antibiotics (ceftazidime, gentamicin, and ciprofloxacin) with 5× MIC for another 24 h and untreated was used as control. Cell viability was measured at t = 0 h, 2 h, 4 h, 6 h, 8 h, and 24 h for each antibiotic treatment according to Soares et al.2019^20^. All experiments were performed in triplicates.

### Cellular redox activity in the biofilm stage

The Biofilm was formed for 24 h and treated with antibiotics as per above mention protocol. After 4 h of antibiotic treatments, the biofilm was once rinsed with 1 ml of 0.85% NaCl to remove planktonic cells and antibiotic. The biofilm was resuspended in 300 µl of 0.85% NaCl and stained with RSG (1 µl) and PI (1 µl of 1:100 diluted) dyes and incubated for 10 min in dark conditions. The untreated biofilm was used as a control. For flow cytometry, the instrument and settings were the same as per above mention protocol.

### Confocal laser scanning microscopy (CLSM) of biofilm

The PCs formation was studied in the biofilm using CLSM. After 5× MIC of antibiotic treatment for 4 h, biofilm was rinsed with 1 ml of 0.85% NaCl to remove planktonic cells and antibiotic. Further, the biofilm was stained with an RSG (1 µl) and PI (1 µl 1:100 diluted) dyes. To remove excess stain from the biofilm a gentle wash with 1 ml of 0.85% NaCl was given. The untreated biofilm was used as a control. The biofilm visualization was done using Carl Zeiss CLSM 780 microscope.

### Gene expression in the biofilm stage

RNA was extracted from the biofilm grown after 4 h of antibiotics treatment. After 4 h of antibiotic treatment with 5X MIC, biofilm was rinsed once with 1 ml of 0.85% NaCl. The remaining biofilm was then pooled in 1 ml of 0.85% NaCl, centrifuged at 8000 rpm for 5 min, and resuspended in 1 ml of 0.85% NaCl. The untreated biofilm was used as a control. The cDNA synthesis and qRT-PCR were performed using the primer listed in Supplementary Table S2 as per above mention protocol for planktonic gene expression.

### Statistical analysis

All experiments were carried out in three biological triplicates. The statistical analysis was carried out using the One-way and Two-way ANOVA test through Graph Prism 9.

## Supplementary Information

Supplementary Information.

## Data Availability

All data generated or analysed during this study are included in this published article [and its supplementary information files].
