# Supplementary material for: Pseudomonas aeruginosa persister cell formation upon antibiotic exposure in planktonic and biofilm state
Source: Sci Rep. 2022 Sep 27;12:16151. doi: 10.1038/s41598-022-20323-3 (PMC9515113; doi:10.1038/s41598-022-20323-3)
Supplement: Supplementary file 1 — Supplementary Information. [file 41598_2022_20323_MOESM1_ESM.docx]

**Supplementary Figure S1: Biofilm quantification**. The biofilm quantification of PAO1(used as reference strain), TP-10, and TP-11 isolate were done using crystal violet assay. The biofilm was formed in the 96-well plate in LB medium for 24 h and the next biofilm was quantified using crystal violet assay. Only LB was used as control. The experiment was performed in three biological triplicates. The one-way ANOVA Tukey test was used in the statistical analysis, and the results were ns*P > 0.05, *P <0.05, and **<P 0.01.

**Supplementary Table S1:** MIC of *P. aeruginosa* isolates

**Supplementary Table S2:** Primers used in the study for the quantification of the gene expression


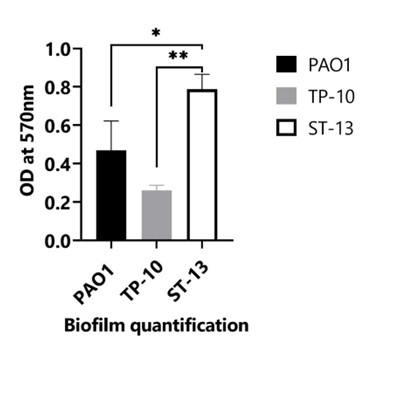


**Supplementary Figure S1:** Biofilm quantification

**Supplementary Table S1:** MIC of P. aeruginosa isolates

CAZ: ceftazidime; GEN: Gentamicin; CIP: Ciprofloxacin Clinical breakpoints for antibiotics according to CSLI standards were ≤8 µg/mL (S), 16 µg/mL (I) ≥32 µg/mL (R) for ceftazidime (CAZ); ≤4 µg/mL (S), 8 µg/mL (I) ≥16 µg/mL (R) for gentamicin (GEN); were ≤1 µg/mL (S), 2 µg/mL (I) ≥4 µg/mL (R) for ciprofloxacin (CIP); R, resistant strains; S, Sensitive strains; I, Intermediate

| **Sample** | **MIC (mg/L)** | | |
| --- | --- | --- | --- |
|  | **CAZ** | **GEN** | **CIP** |
| **PAO1** | 1 (S) | 0.5 (S) | 0.5 (S) |
| **TP-10** | 0.5 (S) | 0.5 (S) | 0.0625 (S) |
| **ST-13** | 2 (S) | 1 (S) | 8 (R) |

**Supplementary Table S2:** Primers used in the study for the quantification of the gene expression

| Function and Primer | Nucleotide sequence (5’-3’) |
| --- | --- |
| RpoD F | GGGCGAAGAAGAAATGGTC |
| RpoD R | CAGGTGGCGTAGGTGGAGAA |
| RelA F | GGCTACATCACGCGTGGG |
| RelA R | CCGCTCATTGAGCAGCAC |
| SpoT F | CGTTCAACGAGATCATGGACG |
| SpoT R | GGTATGCAGCGACTGGTAGC |
| HigA F | CGCGATGAGTTTCTGATGGAG |
| HigA R | CCAGAACTGAGCGGACG |
| HigB F | TTTGAGACGGGTCTTTCG |
| HigB R | TGATGCTATGTTGGCCC |
| Lon F | TATCTCGCCGTGCAAAAGC |
| Lon R | AGCCGATATAGGTACGACGG |
